# Transcriptional analyses of adult and pediatric adamantinomatous craniopharyngioma reveals similar expression signatures regarding potential therapeutic targets

**DOI:** 10.1186/s40478-020-00939-0

**Published:** 2020-05-13

**Authors:** Eric Prince, Ros Whelan, Andrew Donson, Susan Staulcup, Astrid Hengartner, Trinka Vijmasi, Chibueze Agwu, Kevin O. Lillehei, Nicholas K. Foreman, James M. Johnston, Luca Massimi, Richard C. E. Anderson, Mark M. Souweidane, Robert P. Naftel, David D. Limbrick, Gerald Grant, Toba N. Niazi, Roy Dudley, Lindsay Kilburn, Eric M. Jackson, George I. Jallo, Kevin Ginn, Amy Smith, Joshua J. Chern, Amy Lee, Annie Drapeau, Mark D. Krieger, Michael H. Handler, Todd C. Hankinson, on behalf of the Advancing Treatment for Pediatric Craniopharyngioma Consortium

**Affiliations:** 1 Division of Pediatric Neurosurgery, Children’s Hospital Colorado, Aurora, USA; 2 Morgan Adams Foundation Pediatric Brain Tumor Research Program, Aurora, USA; 3 Department of Neurosurgery, University of Colorado School of Medicine, Aurora, USA; 4 Division of Pediatric Neurooncology, Children’s Hospital Colorado, Aurora, USA; 5 Washington University in St. Louis, St. Louis, USA; 6 Division of Pediatric Neurosurgery, Department of Neurosurgery, University of Alabama at Birmhingham, Birmingham, USA; 7 Neurochirurgia Pediatrica, Fondazione Policlinico Universitario A. Gemelli IRCCS, Roma, Italy; 8 Istituto di Neurochirurgia, Università Cattolica del Sacro Cuore, Roma, Italy; 9 Department of Neurosurgery, Columbia University, Morgan Stanley Children’s Hospital of NewYork-Presbyterian, New York, USA; 10 Department of Neurosurgery, Memorial Sloan Kettering Cancer Center, New York, USA; 11 Department of Neurological Surgery, Weill Cornell Medical College, New York, USA; 12 Department of Neurological Surgery, Vanderbilt University Medical Center, Monroe Carell Jr. Children’s Hospital at Vanderbilt, Nashville, USA; 13 Department of Pediatrics, Washington University School of Medicine, St. Louis, USA; 14 Department of Neurosurgery, Washington University School of Medicine, St. Louis, USA; 15 Department of Pediatric Neurosurgery, Lucile Packard Children’s Hospital at Stanford University, Palo Alto, USA; 16 Department of Pediatric Neurosurgery, Nicklaus Children’s Hospital, Miami, USA; 17 Department of Neurosurgery, McGill University, Montreal, Canada; 18 Children’s National Health System, Center for Cancer and Blood Disorders, Washington, DC USA; 19 Children’s National Health System, Brain Tumor Institute, Washington, DC USA; 20 Department of Neurosurgery, Johns Hopkins University School of Medicine, Baltimore, USA; 21 Johns Hopkins All Children’s Hospital, Institute of Brain Protection Sciences, St Petersburg, USA; 22 The Division of Pediatric Hematology and Oncology, the Department of Pediatrics, Children’s Mercy Hospital, Kansas City, USA; 23 Department of Pediatric Hematology-Oncology, Arnold Palmer Hospital, Orlando, USA; 24 Departments of Pediatrics and Neurosurgery, Emory University School of Medicine, Atlanta, USA; 25 Department of Pediatric Neurosurgery, Children’s Healthcare of Atlanta, Atlanta, USA; 26 Department of Pediatric Neurosurgery, Seattle Children’s Hospital, Seattle, USA; 27 Department of Neurological Surgery, University of Washington School of Medicine, Seattle, USA; 28 Division of Pediatric Neurosurgery, Nationwide Children’s Hospital, Columbus, USA; 29 Department of Neurosurgery, Children’s Hospital Los Angeles, Los Angeles, USA

**Keywords:** Adamantinomatous Craniopharyngioma, Transcriptional analysis, Age-related therapy, Pediatric Craniopharyngioma, Suprasellar tumor

## Abstract

Adamantinomatous craniopharyngioma (ACP) is a biologically benign but clinically aggressive lesion that has a significant impact on quality of life. The incidence of the disease has a bimodal distribution, with peaks occurring in children and older adults. Our group previously published the results of a transcriptome analysis of pediatric ACPs that identified several genes that were consistently overexpressed relative to other pediatric brain tumors and normal tissue. We now present the results of a transcriptome analysis comparing pediatric to adult ACP to identify biological differences between these groups that may provide novel therapeutic insights or support the assertion that potential therapies identified through the study of pediatric ACP may also have a role in adult ACP. Using our compiled transcriptome dataset of 27 pediatric and 9 adult ACPs, obtained through the Advancing Treatment for Pediatric Craniopharyngioma Consortium, we interrogated potential age-related transcriptional differences using several rigorous mathematical analyses. These included: canonical differential expression analysis; divisive, agglomerative, and probabilistic based hierarchical clustering; information theory based characterizations; and the deep learning approach, HD Spot. Our work indicates that there is no therapeutically relevant difference in ACP gene expression based on age. As such, potential therapeutic targets identified in pediatric ACP are also likely to have relvance for adult patients.

## Introduction

Adamantinomatous craniopharyngioma (ACP) is a histologically benign brain tumor that arises in the sellar/suprasellar region. Despite being a WHO grade I lesion, ACP often follows a clinically aggressive course and has been associated with the worst quality of life outcomes of any pediatric brain tumor [1]. Unlike its histological counterpart, Papillary Craniopharyngioma (PCP), ACP is characterized by bimodal incidence, with spikes during childhood and adulthood [2, 3]. Also unlike PCP, for which promising therapies targeting the BRAF^v600e^ mutation are in clinical trials, ACP is known to harbor only a mutation in the *CTNNB1* gene, leading to a failure of normal β-Catenin degradation, with resultant cytoplasmic and nuclear accumulation in a subset of cells, where it can act as a transcription factor that may promote ACP pathogenesis. Unfortunately, this understanding has not yet led to the introduction of effective targeted therapies against ACP. As such, current therapies consist primarily of surgery, radiation and intracystic treatments. While downstream effects of the *CTNNB1* mutation appear to be the most likely driver mutation in ACP, it is likely that tumorigenesis is multifactorial. Recent studies of the human ACP transcriptome, using both microarray [4] and RNA sequencing [5], have identified potential novel therapeutic targets. However, this work has focused exclusively on pediatric tumors, leaving open the question of whether these potential therapies could be relevant in adult patients, who represent approximately 69% of all patients with ACP [2, 3].

This work sought to investigate, using bulk RNA sequencing of adult and pediatric ACP, whether there is evidence that patients with ACP from different age cohorts could be expected to respond to the same targeted therapeutic strategies. In order to identify even subtle differences in the transcriptome profile of the 2 tissue populations, we conducted a highly rigorous mathematical analysis that considered the potential for both linear and non-linear age group-related dependencies within the data.

## Materials and methods

### Tumor samples

A total of 36 craniopharyngioma tumor samples, as diagnosed by surgical pathology, were included in this study (Table S1). Twenty-seven were acquired during surgery for pediatric patients (age at surgery < 18 years; mean = 8.4 years, median = 8.0 years), and 9 from adults (age at surgery ≥18 years; mean = 47.9 years, median = 44.0 years). The pediatric specimens were obtained from patients who underwent surgery at member institutions of the Advancing Treatment for Pediatric Craniopharyngioma (ATPC) consortium. Adult specimens were obtained from University of Colorado Hospital and from the University of Alabama, Birmingham. Tumor samples were snap-frozen in liquid nitrogen in the operating room and subsequently stored in freezers at − 80 °C. For transport between institutions, samples were packaged on dry ice, shipped via overnight courier, and immediately placed in a freezer at − 80 °C.

### RNA extraction and sequencing

RNA was extracted from snap-frozen samples using the Allprep DNA/RNA Kit (QIAGEN®, Maryland, USA). The quality of the isolated mRNA was determined via DNA analysis ScreenTape (Aligent Technologies). Samples that passed quality control were used to generate cDNA libraries using the Illumina TruSeq Stranded mRNA Sample Prep Kit. RNA sequencing was carried out using the Illumina HiSeq4000 platform with paired-end reads (2 × 151). On average, 40 million reads were collected for each sample and outputted to FASTQ files. Sequencing reads were subjected to adapter-trimming and quality control using the Trimmomatic package [6]. Files were mapped to the GRCh38 genome (v33) and subsequently sorted to yield BAM files using the standard STAR pipeline [7]. BAM files were converted to feature counts using the R Bioconductor package RSubread [8]. Normal pituitary RNA sequencing data was obtained from the GTEx portal (www.gtexportal.org).

### Analysis methodology

Due to the possibility that adult and pediatric transcription patterns would be distinguished by only subtle expression differences, we chose a multimodal bioinformatic approach. This was designed to assess for both linear and non-linear relationships, but also to insulate the statistical analysis from potentially false assumptions.

### Canonical differential expression analysis

Feature counts for transcriptomes of adult and pediatric ACP samples were analytically processed using a standard DESeq2 protocol [9]. As the experiment intended to rule out differences in gene expression between the 2 age groups, an Independent Hypothesis Weighted (IHW) *p*-value threshold of 0.1 was utilized to filter transcripts identified as differentially expressed. Results were visualized using a Mass Action (MA) plot and a Euclidean Distance Matrix was employed to serve as a qualitative foundation from which to make statistical methodology arguments downstream.

**Fig. 1:**
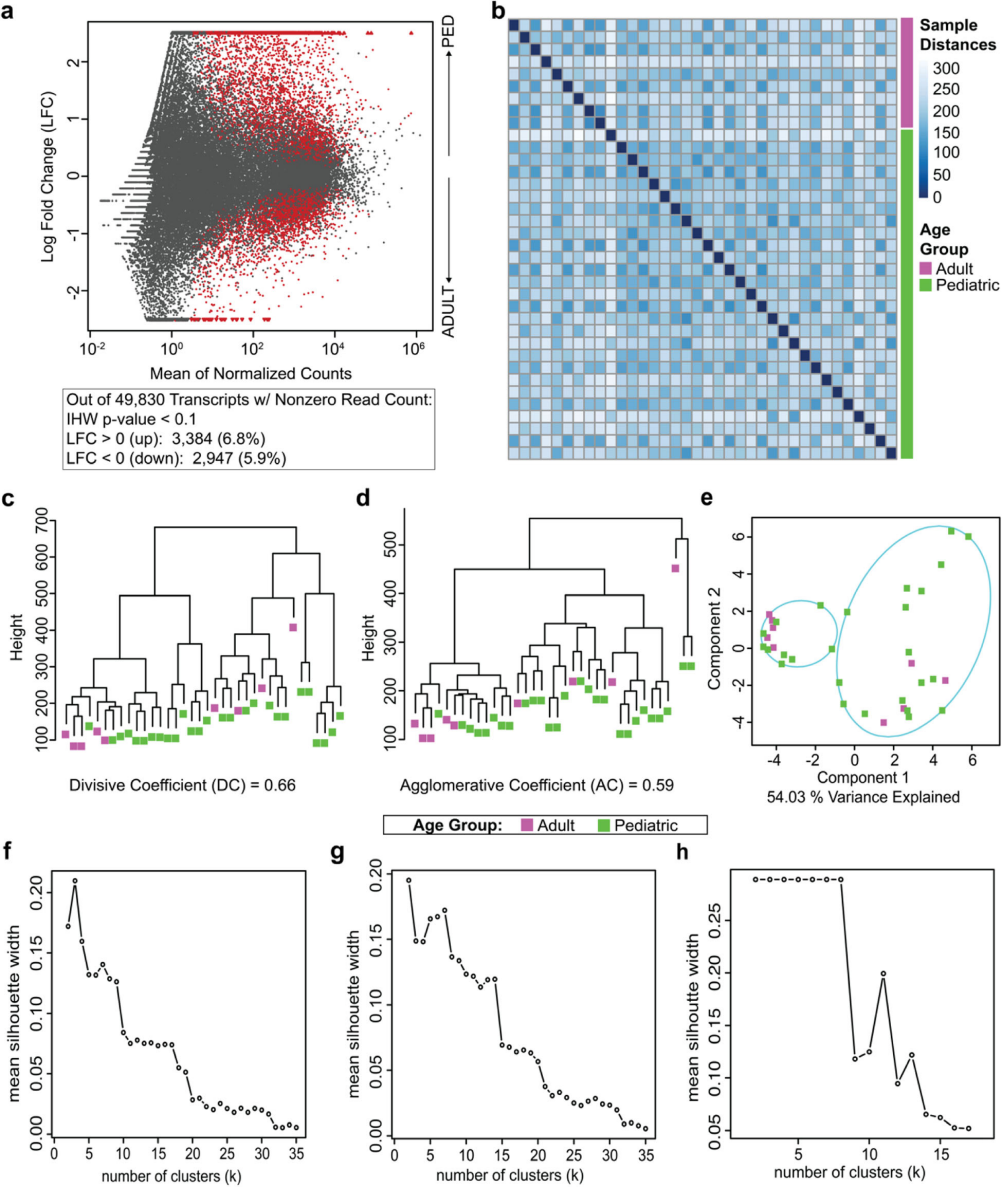
Global Transcriptional Profiling of Adult and Pediatric ACP Samples. **a** MA-plot visualizing transcripts indicating significant (Independent Hypothesis Weighting (IHW) adjusted *p*-value < 0.1; red) and insignificant genes (black) as determined in differential expression analysis; transcripts enriched relative to pediatric patients are log fold change (LFC) up (> 0) and transcripts enriched relative to adult patients are LFC down (< 0). **b** Euclidean sample distance matrix without clustering demonstrated the relative heterogeneity across all sample groups from a global transcriptional expression perspective. **c**-**e** Clustering paradigms utilized in dataset exploration. **c** Dendrogram yielded from the DIvisive ANAlysis (DIANA) hierarchical clustering algorithm. **d** Dendrogram produced by the AGlomerative NESting (AGNES) hierarchical clustering algorithm using complete linkage. **e** Clustering partitions and group ellipsoids generated by the fuzzy analysis (FANNY) probabilistic k-centroid technique. **f-h** Silhouette plots depicting mean silhouette width across a range of numbers of clusters. Possible silhouette values are within [− 1,1] where a value of 1 indicates the cluster member is most-closely related to the members within that cluster and dissimilar to those outside of the cluster. As values approach − 1, the opposite is true which indicates the cluster member is an outlier within the cluster. **f** Silhouette plot generated from the DIANA clustering presented in 1c. **g** Silhouette plot produced by the AGNES algorithm output presented in 1d. **h** Silhouette plot yielded by the FANNY algorithm results visualized in 1e

Divisive, agglomerative, and probabilistic clustering was performed using the DIANA, AGNES, and FANNY functions in the cluster R package [10], respectively. AGNES was implemented with the complete-linkage method. Complete-linkage clustering groups variables by their most dissimilar members and also avoids combining clusters with highly similar elements (as opposed to single-linkage). As shown below in Fig. 1b, we expect no linear dissimilarity between transcriptomes relative to age group, making complete-linkage an appropriate clustering strategy. As subtle differences in the gene expression patterns could be expected, complete-linkage was employed due to its superiority over a single-linkage approach in handling data groups that closely neighbor one another. DIANA and AGNES calculate metrics for cluster organization known as the Divisive Coefficient (DC) and Agglomerative Coefficient (AC), respectively. These coefficients range between 0 and 1, and indicate the precision with which the dendrogram describes the data, with 1 being the most ideal structure and 0 being the worst. As FANNY is not a hierarchical algorithm, and is visualized in principal component space, the percent of variance explained serves as a surrogate value for AC and DC (i.e., a larger percent variance explained value indicates how well a datasets information is represented in the decomposed space).

Although cluster analysis is a primarily descriptive mathematical technique, we calculated the silhouette width as a means to make a quantitative comparison of the differential expression data. Silhouette widths range between − 1 and 1. A value of 1 indicates that the cluster members are closely related to the other members of the cluster, and not to members outside the cluster. A value of − 1 suggests the cluster member is an outlier within the cluster and is actually more representative of other clusters. The mean Silhouette Value was calculated for a range of cluster numbers for the DIANA, AGNES, and FANNY algorithms (Fig. 1f, g, h, respectively).

### Information theory analysis

In order to extend the analysis beyond the linear methodology utilized in the canonical analysis, we calculated the Kullback-Leibler (KL) divergence (*D*_KL_) and Maximal Information Coefficient (MIC). This was necessary because non-linear transcriptional relationships (i.e., dynamic networks of transcripts) between age groups could exist and are likely abundant, as seen by the development of numerous pathway-based analytical tools (e.g., GSEA, GO-terms, Reactome, etc.). While these types of modules are interesting they often require predefined lists. However, utilizing information theory allows us to inspect potential networks in the absence of prior known networks, and also reduces analytical bias.

*D*_KL_ is a mathematical approach to identify differences between data distributions through non-linear integration. While this is most commonly used to compare machine learning outputs to ground truth distributions, we employed it by treating one distribution (e.g., pediatric) as the predicted distribution and the other distribution (e.g., adult) as the target distribution (e.g. “ground truth”). This scenario effectively asks the following question: given the expression distribution of the adult cohort, how well can the distribution of the pediatric cohort be predicted. To calculate D_KL_, raw feature counts generated by RSubread were first class-balanced using the Synthetic Minority Oversampling Technique (SMOTE) [11] via the python imblearn package [12]. *D*_KL_ values were then calculated for the synthetically balanced dataset using the python scipy stats module.

MIC is used to measure the strength of any linear and/or non-linear relationship between two variables. This is accomplished by binning continuous variables and iteratively calculating mutual information (i.e., the dependence between variables) to identify the maxima. By plotting MIC against Pearson’s R the magnitude of the relationship between the variables can be visualized (MIC = 0 indicates no relationship; MIC = 1 indicates highly related), as can the nature of the relationship (linear vs. non-linear). MIC was calculated using the python minepy module [13].

### HD spot analysis

Raw feature counts derived from RSubread were submitted to the HD Spot algorithm [14]. Due to the substantial imbalance of data classes, the HD Spot algorithm was optimized with respect to maximizing the area under the precision-recall (AUPR) curve. HD Spot developed a classifier that achieved an average AUPR value of 1.00 over 5-fold cross-validation and subsequently determined the mean absolute Shapley value for each transcript. Shapley values can conceptually be understood as importance scores. In this context, a higher Shapley value means a transcript is more important in determining the age group from which the sample was taken. The top 50 transcripts ranked by Shapley value and the list of 20 previously identified therapeutic targets were then submitted for Metascape [15] express analysis to explore potential ontologic connections between gene sets using GO terms.

## Results

### Canonical differential expression analysis reveals no therapeutically relevant distinction between adult and pediatric ACP specimens

Overall, 87.3% of the transcripts interrogated were insignificant (IHW *p*-value > 0.1) and slightly more transcripts enriched within the pediatric population versus the adult population (6.8% versus 5.9%, respectively, Fig. 1a). Considerable heterogeneity between all samples (irrespective of age group) was identified with the Euclidean distance matrix (Fig. 1b). Both the agglomerative and divisive clustering algorithms demonstrated reliable data organization (Fig. 1c-d; Divisive Coefficient [DC] = 0.66, Agglomerative Coefficient [AC] = 0.59) as well as the absence of distinct clusters between the adult and pediatric ACP samples. Congruently, probabilistic analysis in 2D principal component space, using FANNY, depicted nearly 70 % of the data variance (69.93%, Fig. 1e) and failed to partition the data into groups based on age cohort. Further analyses of these clustering approaches, using silhouette plots to quantify clustering integrity, demonstrated that *k* = 2 yields the optimal clustering (Fig. 1f-h). However, all silhouette widths are close to 0 (maximum ≈ 0.30), indicating that clusters are not unique from one another and cluster memberships could easily be randomized another way. When examined as a whole, agglomerative, divisive, and probabilistic clustering algorithms yield consistent and well-organized results, indicating that the transcriptomes of ACP specimens do not cluster based on the age of the patient from which they were obtained.

**Fig. 2:**
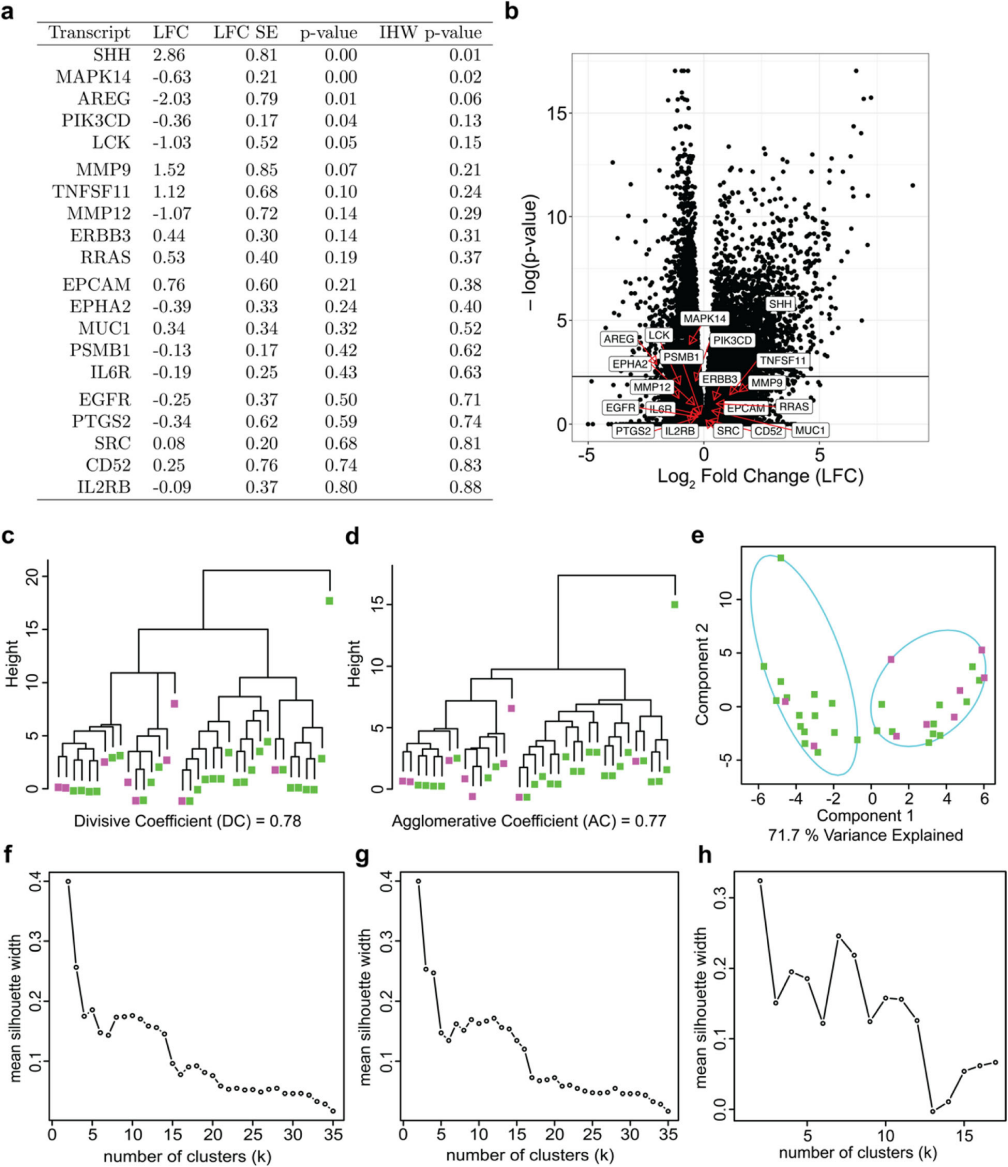
Transcriptional Profiling of Previously Identified Therapeutic Targets. **a** Previously identified therapeutic targets with transcriptions fold-change metrics for adult versus pediatric samples. A positive fold-change indicates enrichment in pediatric patients, and conversely a negative value indicates enrichment in adult patients. **b** Volcano plot for all transcripts with previously identified targets indicated by red arrows. The solid black horizontal line at y = 1 indicates the threshold for *p*-value significance (*p* < 0.1; y = −log(0.1) = 1). **c**-**e** Clustering paradigms utilized in dataset exploration. **c** Dendrogram yielded from the DIvisive ANAlysis (DIANA) hierarchical clustering algorithm with respect to only the twenty previously identified targets. **d** Dendrogram produced by the AGlomerative NESting (AGNES) hierarchical clustering algorithm using complete linkage with respect to only the twenty previously identified targets. **e** Clustering partitions and group ellipsoids generated by the fuzzy analysis (FANNY) probabilistic k-centroid technique with respect to only the twenty previously identified targets. **f-h** Silhouette plots depicting mean silhouette width across a range of numbers of clusters. **f** Silhouette plot generated from the DIANA clustering presented in 2c. **g** Silhouette plot produced by the AGNES algorithm output presented in 2d. **h** Silhouette plot yielded by the FANNY algorithm results visualized in 2e

Our group previously identified a group of twenty potentially targetable transcripts within pediatric ACP [4]. The primary clinical question underlying this research is to determine whether initial treatment strategies against ACP should be differentiated based on age at diagnosis. Accordingly, we validated these targets in the current dataset compared to normal pituitary (Figure S1) and created a subset of the transcriptome data focusing on these twenty targets to assess differential expression status (Fig. 2a and b). Of the previously identified potential targets, three genes were significant (IHW *p*-value < 0.1): SHH, MAPK14, and AREG. Next, this data subset was subjected to the same divisive, agglomerative, and probabilistic clustering protocols (Fig. 2c-e) as the total dataset above. There was slight improvement in data organization (i.e., an increase in clustering coefficients; DC = 0.78, and AC = 0.77, relative to 0.66 and 0.59 when analyzing the full transcriptome). Additionally, *k* = 2 remained the superior cluster count but higher *k* values improved relative to the clustering performance of the full transcriptome (Fig. 2f-h). Although four genes were significantly differentially expressed, the data subset remained linearly undifferentiable based on age. In other words, these results indicate that there is no direct linear relationship between transcriptional profiles and age group at diagnosis.

### Information theory and machine learning techniques identify a cohort of transcripts relevant to differentiating adult and pediatric patients

**Fig. 3:**
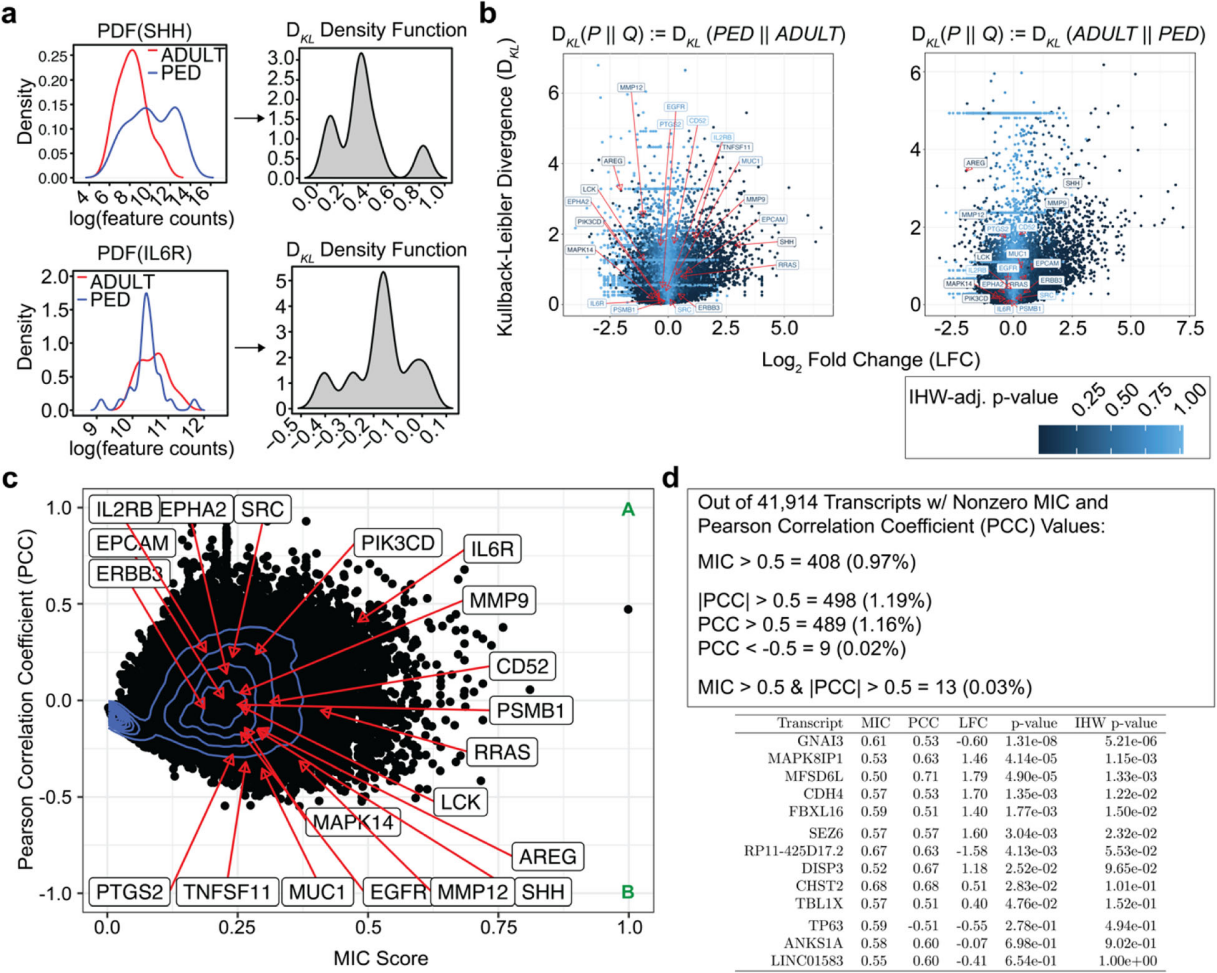
Information Theory-Based Analysis Suggests Majority of Genes Have Minimal Linear and Non-linear Relationships with Age Groups. **a** Kullback-Leibler (KL) divergence representation of SHH (top) and IL6R (bottom) distributions for adult and pediatric samples. **b** KL-divergence versus Log Fold Change plot, with previously identified therapeutic targets overlaid and all points colored by IHW-adjusted *p*-value, demonstrating relationship between calculated LFC and informational differences. Higher KL-Divergence values indicate that a gene has informational difference between age groups. As KL-Divergence is an asymmetric method, the scenario of having a pediatric prior (left) and an adult prior (right) are both shown. **c** Pearson Correlation Coefficient (PCC) vs Maximal Information Criterion (MIC) plot with previously identified targets overlaid. MIC scores the strength of a relationship from 0 (no relationship) to 1 (noise-free relationship) for genes between the age groups. Points A and B on the graph represent where genes should fall if they have a strong direct or inverse linear relationship with age groups. Values that have low PCC and high MIC scores indicate genes with non-linear (i.e. dynamic; one-vs-many) relationships. **d** Summary statistics of where genes lie on the PCC vs MIC plot along with their respective differential expression values as visualized in (**b**)

In the present context, Kullback-Leibler divergence (*D*_KL_) represents the difference between transcript expression profiles of the two age groups (Fig. 3a). *D*_KL_ is asymmetric, so we visualize both directions of the relationship (i.e., Pediatrics to Adults, and Adults to Pediatrics; Fig. 3b). These results demonstrate that qualitatively the global transcriptomes of both age groups are informationally similar. Of the four differentially expressed therapeutic targets, SHH and AREG demonstrate the greatest informational difference (D_*KL*_ ≈ 3) although still less than 50 % of maximum (D_*KL*_ ≈ 6) informational differences (i.e., they are not among the top contributors for informational difference between age groups).

We analytically assed both linear and non-linear relationships by comparing the MIC versus Pearson’s R (Fig. 3c). From this we determined that of the 41,914 transcripts interrogated only 0.03% (*N* = 13) genes maintain expression levels that differ based on age group (Fig. 3d). The encoded genes include a long intergenic non-protein coding gene, innate immune system related genes, a chromosomal open reading frame, and a keratin metabolism related gene. When cross-checked with canonical differential analysis methods, four genes (FBXL16, COG8, CHST2, and TMCC2) were statistically significant as determined by *p*-value. However, Log Fold Change (LFC) magnitudes were less than 1 for all but FBXL16 (LFC = 1.38). Of the seven genes identified, none are currently of therapeutic interest.

### Ontology assessment of deep learning identified age group-differentiable transcripts

**Fig. 4:**
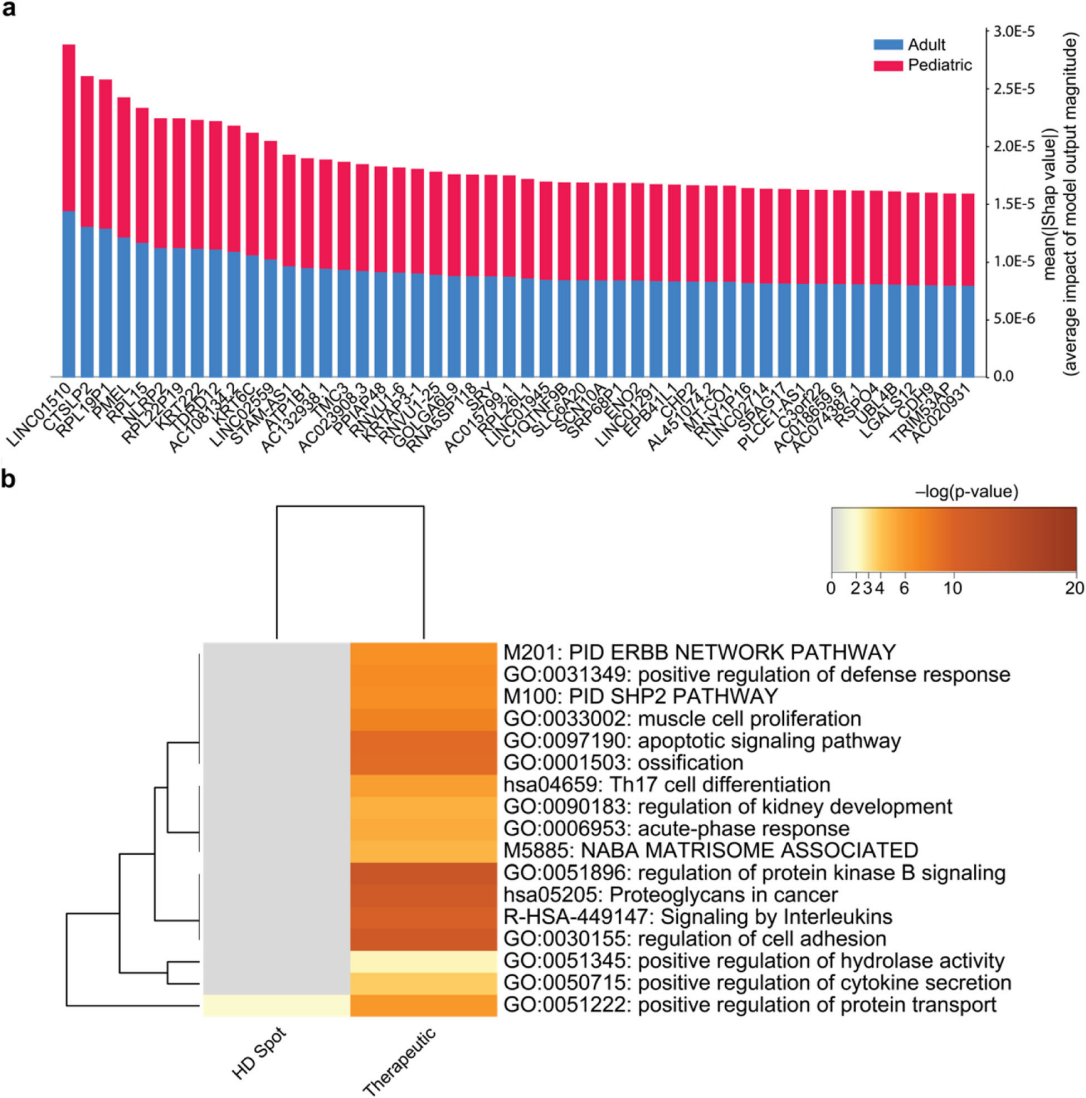
Deep Learning Approach HD Spot Identifies Genes Related to Adult and Pediatric Cohort Differences From Raw Feature Counts. **a** Summary plot of the top 50 genes identified by HD Spot as being the most important in separating adult and pediatric ACP transcriptomes. **b** Heatmap of GO terms found to be enriched in Metascape analysis comparing HD Spot-identified and the 20 previously identified therapeutic targets. Heatmap color represents ontology term enrichment

Following classification using the HD Spot algorithm, the 50 (0.20%) transcripts with the highest Shapley values (Fig. 4a) and the 20 previously identified potential therapeutic targets were examined using a Metascape analysis (Fig. 4b). HD Spot identified primarily pseudogenes, novel transcripts, and non-coding transcripts as most differentiable between age groups. Only one ontology group (GO:0051222) is enriched in HD spot analysis and it is also enriched (though more significantly) with respect to the therapeutic targets. The lack of enrichment returned from Metascape analysis reinforces that the differential genes identified are not well studied and are currently not of therapeutic interest. Importantly, HD Spot achieved a mean 5-fold cross-validation area under precision-recall curve value of 0.97, indicating that the derived classifier was well optimized. In total, these findings indicate that while there may be underlying transcriptional difference for ACP pathogensis relative to pediatric and adult patients, current therapeutic targets do not maintain age-dependent linear or non-linear expression signatures.

## Discussion

Using a thorough comparative bioinformatic analysis following bulk tissue RNA sequencing of pediatric and adult ACP tissue, we identified no gene expression differences that have implications regarding potential therapies. This implies that potential therapies identified or initially tested in either the adult or pediatric population will have relevance in the other age group. To our knowledge this work consists of the largest analysed cohort of ACP transcriptome data to date.

Canonical differential expression techniques demonstrated no clear differences between age groups. At the global level, only 12 and 13% of transcripts yielded a Log Fold Change (LFC) with an IHW-adjusted *p*-value < 0.1. Using divisive, agglomerative, and probabilistic hierarchical clustering approaches, we observed that while clustering was at a relative optimum with *k* = 2 clusters, cluster memberships were arbitrary (mean silhouette widths < 0.35) and contained a mixture of age groups in each branch. In the context of the twenty previously identified potential therapeutic targets, we found that only SHH, AREG, and MAPK14 were differentially expressed with adjusted *p*-value < 0.1. Analysis of these 20 transcripts demonstrated the same clustering performance results that were observed with analysis of the entire transcriptome. Specifically, adult and pediatric patients did not cluster uniquely and inter-cluster members could arbitrarily be replaced by intra-cluster members.

The findings from the canonical techniques were confirmed through further analysis using information theory principles. This demonstrated that 99.97% of the transcriptome has no expression level relationship with the age group of the patient. The remaining 0.03% was composed of a mixed collection of transcripts that have not been implicated in tumor pathogenesis or therapy, and LFCs were modest.

Next, we sought to qualitatively examine non-linear transcriptional relationships to age group by utilizing KL-divergence. This again demonstrated that the global transcriptome profile for pediatric ACP differs very little from that of adult ACP. Quantitative assessment of linear and non-linear relationships using MIC vs. Pearson’s R plots revealed that over 99% of transcripts do not possess strong relationships of any type to age group. Importantly, the required synthetic expansion of the dataset (see Methods) in order to calculate *D*_KL_ and MIC would be expected to artificially magnify any data relationships. Therefore, the lack of any clear relationship between transcript expression and age group in this context further supports the assertion that the adult and pediatric transcriptomes lack therapeutically relevant differeces.

The threshold selected to distinguish adult from pediatric ACP (age 18 years) may be considered arbitrary. However, the bimodal age distribution of ACP incidence includes a nadir between age 20 and age 39. This implies that there may be a distinction, on some level, between the tumors that present before and after this window. The age threshold of 18 years has further clinical significance because it relates to the clinical environment (pediatric vs. adult) in which a patient may receive care. Furthermore, by leveraging the deep learning technique HD Spot, we identified genes, in a manner free of human-interpreter bias, that have age group dependent expression profiles. HD Spot accurately discriminated between pediatric and adult transcriptomes. The top 50 genes contributing to this classification were primarily comprised of genomic features that are currently poorly understood (e.g., pseudogenes, novel transcripts, etc.). Through utilizing classical genomic analysis protocols, information theoretic models, and deep learning this work rigorously interrogated linear and non-linear relationships to demonstrate that, at the transcript level, there is no evidence to differentiate therapy between adult and pediatric ACP patients strictly based on age at diagnosis.

### Limitations

Despite the rigorous characterization of the transcriptional relationship between pediatric and adult ACP tumor tissue, our findings are limited by the fact that only RNA expression profiles were considered. This provides a specific window into the genomic landscape. It does not provide thorough insight into potential differences in the epigenetic environment or extracellular milieu between pediatric and adult ACP. Furthermore, the transcriptome data analyzed was generated through bulk RNA sequencing. Given heterogeneous nature of ACP tissue, bulk sequencing is likely to lose some information, which may be explored in more detail with techniques such as single cell RNAseq, RNA mutation analysis or protein-level quantification. Lastly, there is also still potential that an even larger dataset contrasting these age groups (which to our knowledge does not exist) may still reveal more therapeutically important differences. Such studies may identify biological distinctions that could guide therapeutic intervention, even if this is at the level of second or third line therapy.

## Conclusions

Analysis using multiple bioinformatic techniques, including the Deep Learning method, HD Spot, indicates that there is no therapeutically relevant distinction between ACP tissuein adult and pediatric patient. This implies that the identification of biologically-guided therapeutic targets, and the potential clinical translation of such targets in either group of patients may also be relevant in the other. As such, recent and future findings regarding ACP may be applied for the benefit of a larger group of patients. Future work including more in depth study of RNA features, protein expression and extracellular characteristics of these tumors will be necessary.

## Supplementary information

**Additional file 1: Figure S1.** Log fold change versus *p*-value for therapeutic targets across ACP age groups compared to GTEx normal pituitary dataset. **Table S1.** Dataset Demographics and CTNNB1 Mutation Status. Hyphen values indicate data not available. WT: Wild Type; Age at Dx: Age at Diagnosis.

## Data Availability

The datasets during and/or analyzed during the current study is available from the corresponding author on reasonable request.
