# Supplementary material for: Transcriptional analyses of adult and pediatric adamantinomatous craniopharyngioma reveals similar expression signatures regarding potential therapeutic targets
Source: Acta Neuropathol Commun. 2020 May 13;8:68. doi: 10.1186/s40478-020-00939-0 (PMC7222517; doi:10.1186/s40478-020-00939-0)

**Supplemental Data**

**
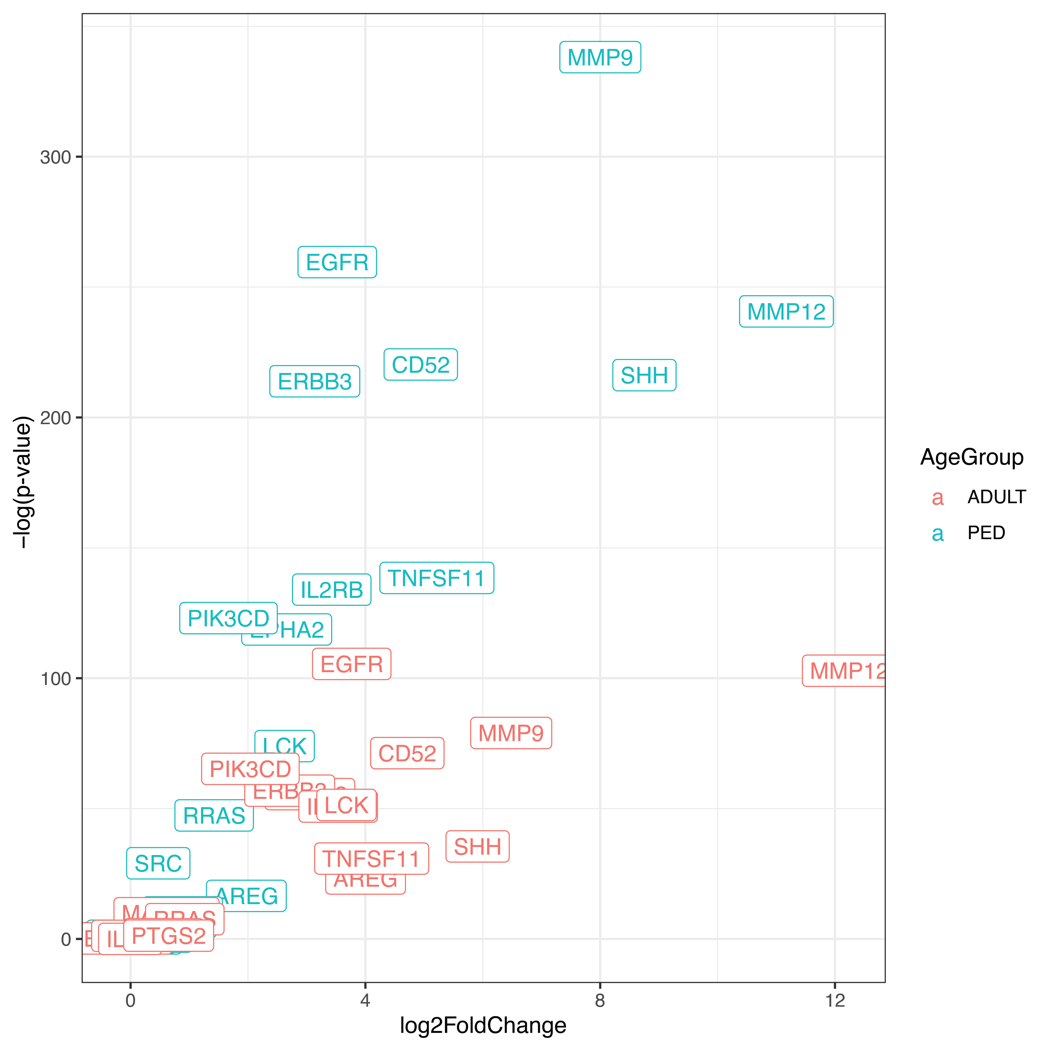
**

**Figure S1.** Log fold change versus p-value for therapeutic targets across ACP age groups compared to GTEx normal pituitary dataset.

**Table S1.** Dataset Demographics and CTNNB1 Mutation Status. Hyphen values indicate data not available. WT: Wild Type; Age at Dx: Age at Diagnosis.


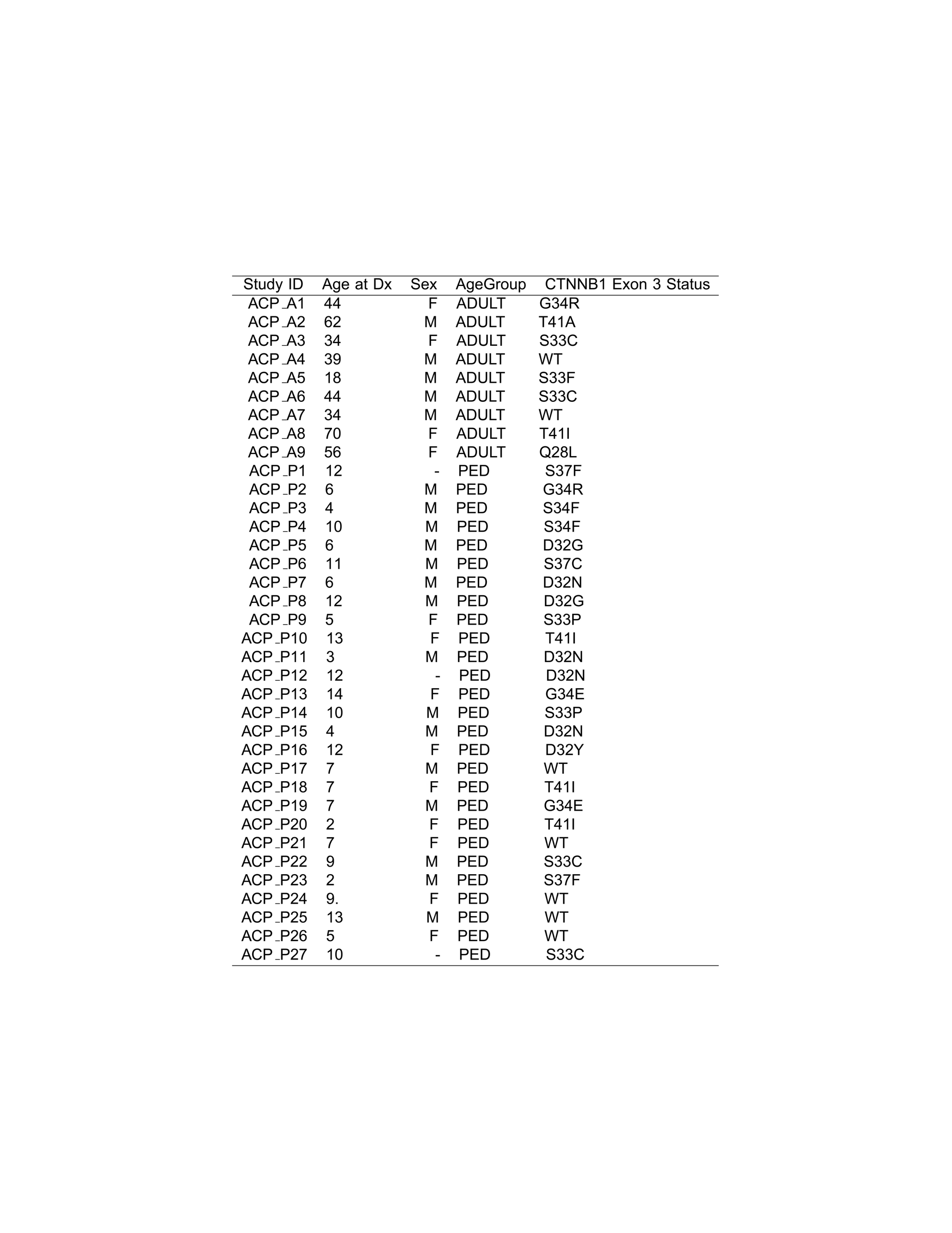

Supplement: Supplementary file 1 — Additional file 1: Figure S1. Log fold change versus p-value for therapeutic targets across ACP age groups compared to GTEx normal pituitary dataset. Table S1. Dataset Demographics and CTNNB1 Mutation Status. Hyphen values indicate data not available. WT: Wild Type; Age at Dx: Age at Diagnosis. [file 40478_2020_939_MOESM1_ESM.docx]
